# Minimally Invasive Endovascular Administration for Targeted PLGA Nanoparticles Delivery to Brain, Salivary Glands, Kidney and Lower Limbs

**DOI:** 10.3390/pharmaceutics18010085

**Published:** 2026-01-09

**Authors:** Olga A. Sindeeva, Lyubov I. Kazakova, Alexandra Sain, Olga I. Gusliakova, Oleg A. Kulikov, Daria A. Terentyeva, Irina A. Gololobova, Nikolay A. Pyataev, Gleb B. Sukhorukov

**Affiliations:** 1 Vladimir Zelman Center for Neurobiology and Brain Rehabilitation, Skolkovo Institute of Science and Technology, 30 Bolshoy Boulevard, 121205 Moscow, Russia; alexandra.sain@skoltech.ru (A.S.); olga.gusliakova17@gmail.com (O.I.G.); daria.terentyeva@skoltech.ru (D.A.T.); 2 Life Improvement by Future Technologies (LIFT) Center, 30 Bolshoy Boulevard, 143025 Moscow, Russia; l.kazakova@lift.center; 3 Science Medical Center, Saratov State University, 83 Astrakhanskaya Street, 410012 Saratov, Russia; 4 Institute of Medicine, National Research Ogarev Mordovia State University, 68 Bolshevistskaya Str., 430005 Saransk, Russia; oleg-kulikov-84@mail.ru (O.A.K.); irina.gololobova16@yandex.ru (I.A.G.); pyataevna@mail.ru (N.A.P.); 5 Center for Photonic Science and Engineering, Skolkovo Institute of Science and Technology, 30 Bolshoy Boulevard, 121205 Moscow, Russia

**Keywords:** PLGA nanoparticles, minimally invasive endovascular microsurgery, carotid artery injection, renal artery injection, femoral artery injection, targeted drug delivery, nanomedicine

## Abstract

**Background:** While intravenous administration of nanoparticles (NPs) is effective for targeting the lungs and liver, directing them to other organs and tissues remains challenging. **Methods:** Here, we report alternative administration routes that improve organ-specific accumulation of poly (lactic-co-glycolic acid) (PLGA) NPs (100 nm, negatively charged) loaded with the near-infrared dye Cyanine 7 (Cy7). NP cytotoxicity was evaluated in HEK293, mMSCs, C2C12, L929, and RAW264.7 cells. Hemocompatibility was assessed using WBCs and RBCs. NPs were administered via the tail vein, carotid, renal, and femoral arteries in BALB/c mice. Administration safety was evaluated by laser speckle contrast imaging and histological analysis. NP biodistribution and accumulation were assessed using in vivo and ex vivo fluorescence tomography and confocal microscopy of cryosections. **Results:** PLGA-Cy7 NPs demonstrate low cytotoxicity even at high doses and exhibit good hemocompatibility. Administration of NPs through the mouse carotid, renal, and femoral arteries significantly increases accumulation in the target ipsilateral brain hemisphere (31.7-fold) and salivary glands (28.3-fold), kidney (13.7-fold), and hind paw (3.6-fold), respectively, compared to intravenous administration. Injection of NPs through arteries supplying the target organs and tissues does not result in significant changes in blood flow, morphological alterations, or irreversible embolization of vessels, provided the procedure is performed correctly and the optimal dosage is used. **Conclusions:** These results highlight the potential of intra-arterial delivery of NPs for organ-specific drug targeting, underscoring the synergistic impact of advances in materials science, minimally invasive endovascular surgery, and nanomedicine.

## 1. Introduction

The circulatory system is a unique evolutionary transport pathway that not only delivers nutrients, biologically active molecules, and cells to various organs but also ensures their uniform distribution within tissues. This is why the idea of intravascular targeting of drug delivery systems capable of delayed, prolonged, or controlled drug release remains of considerable interest in nanomedicine [1,2,3,4,5]. Nevertheless, systemic intravenous administration of NPs into the bloodstream remains challenging due to the well-known first-pass effect, which leads to their preferential accumulation in the lungs and liver—organs with a highly developed reticuloendothelial system [6].

Surface modification is commonly employed to improve the targeting of NPs to other organs and tissues because it can significantly improve the interaction of particles with specific cells, as demonstrated in numerous in vitro studies using cell lines [7,8]. This is achieved by incorporating additional functional moieties into the particle structure, followed by chemical conjugation to targeting ligands, typically small biologically active molecules, antibodies, or nucleotide sequences [9,10,11]. Additionally, amphiphilic polymers may be introduced to impart hydrophilic properties to the particles. Nevertheless, any modification of NP properties requires comprehensive safety and biodegradation studies. At the same time, surface modification generally results in an increase in particle size [7,12], with only slight changes in their distribution and circulation time [6].

Minimally invasive endovascular surgery offers opportunities for targeted drug delivery by exploiting the first-pass effect through the direct administration of NPs and other carriers into arteries supplying the target tissue. Despite its established clinical use for vascular implants [13,14], this approach has not yet been translated to the delivery of drug carriers. Experimental evidence regarding the safety and efficacy of arterial delivery remains limited to a small number of studies involving inorganic NPs [15,16,17], polymer NPs [18,19,20], and liposomes [21,22,23,24], largely due to the technical complexity of in vivo procedures requiring specialized microsurgical expertise. Our previous studies demonstrated that arterial administration can markedly alter the biodistribution of polyelectrolyte microcapsules without surface modification [25,26,27]. Targeting 3–5 µm polyarginine–dextran sulfate capsules via the femoral or renal arteries increased delivery efficiency to the hind paw and kidney, respectively [25,26,27,28], with appropriate dosage ensuring both safety and efficacy [26,29,30]. Incorporation of magnetic NPs further enhanced accumulation under an external magnetic field [28,31,32], an effect also reported for brain tumor targeting by independent studies [15]. Nevertheless, not all drug carriers exhibit efficient accumulation following arterial delivery, as demonstrated for whey protein–stabilized microgels [33]. Therefore, the arterial targeting performance of each carrier must be systematically evaluated for specific organs and tissues. Furthermore, despite extensive research on surface modification strategies, the capacity of minimally invasive arterial delivery to enhance nanoparticle uptake in distinct organs remains insufficiently understood.

**Figure 1:**
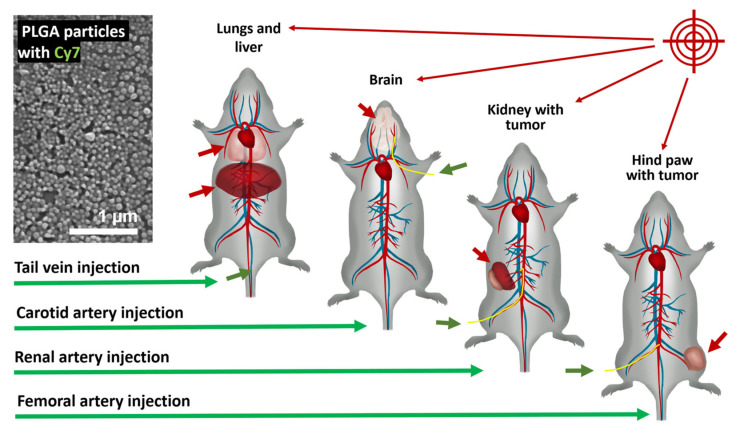
Schematic overview of the experimental design for intra-arterial targeting of NPs in the brain, kidney, and hind paw (green arrows indicate routes of administration; red arrows indicate target organs or tissues).

In this study, we investigated arterial targeting as a minimally invasive endovascular approach for the effective and safe delivery of PLGA NPs, which are widely used in medicine [34,35,36] as carriers for pharmaceutical compounds [36,37]. PLGA NPs differ significantly from the polyelectrolyte microcapsules examined in our previous work in terms of size, composition, physicochemical characteristics, and expected behavior. Here, we synthesized and characterized PLGA particles with a nominal diameter of 100 nm, loaded with Cyanine 7 (Cy7) as both a model cargo and an imaging agent, and evaluated their cytotoxicity and hemocompatibility. NPs were administrated via the carotid, renal, and femoral arteries to target the brain and salivary glands, the kidney bearing an orthotopic renal tumor (Renca), and the hind paw with a murine colorectal carcinoma (CT26) tumor, respectively (Figure 1). We assessed the in vivo and ex vivo biodistribution profiles of the particles in comparison with conventional intravenous administration, as well as their tissue localization and the safety of the proposed approach, using histological analysis and blood flow monitoring.

## 2. Materials and Methods

### 2.1. Reagents

PLGA 50/50 (lactic/glycolic acid, mol%) with a molecular weight of 100–150 kDa and ester-terminated end groups was purchased from Novochem (Tomsk, Russia). The fluorescent dye Cy7 NHS ester was obtained from Lumiprobe (Cockeysville, MD, USA) (cat. no. 65020). Tween 80 (pure EP, cat. no. LC-10954.2, pharmaceutical grade) was purchased from neoFroxx GmbH (Einhausen, Germany). A 0.9% sterile NaCl solution was obtained from Solopharm (Saint Petersburg, Russia). Acetone (analytical grade) was purchased from Rushim (Moscow, Russia). Ultrapure water (Millipore, Molsheim, France) with a resistivity of 18.2 MΩ·cm was used in all experiments. All chemicals were used as received without further purification.

### 2.2. NPs Preparation

PLGA NPs were prepared using a nanoprecipitation technique [38]. For this, 16 mg of PLGA and 0.15 mg of drug were dissolved in 4 mL of acetone. This organic phase was added dropwise into 16 mL of ultrapure water without buffer, containing 20 mg of Tween-80, under moderate stirring (800 rpm) using an IKA C-MAG HS 7 hotplate stirrer (IKA, Staufen, Germany), with the dispersion medium maintained at the boiling point of acetone. Solvent was removed from the colloidal suspension by continued evaporation for 35 min under heating. The resulting particles were collected by centrifugation at 8000 rpm for 30 min (Eppendorf MiniSpin, Hamburg, Germany), washed four times with water using the same procedure, and resuspended in 2 mL of 0.9% saline. The mass yield of the obtained particles was 52 ± 5%. The final particle concentration in the suspension used for the study was 4.2 ± 0.4 mg/mL. Samples were stored protected from light at 4 °C until further use.

### 2.3. NPs Characterization

#### 2.3.1. Determination of Particle Size

The Z-average size, mean diameter, particle size distribution (PDI), and ζ-potential of the NPs were determined by dynamic light scattering (DLS) using a Zetasizer Nano ZS (Malvern, UK). For this purpose, 10 µL of the NP suspension was diluted in 1 mL of ultrapure water, and the resulting dispersion was loaded into a disposable sizing cuvette or a U-shaped cuvette for size and ζ-potential measurements, respectively. No additional filtration of the particles was performed after preparation. During analysis, the refractive index of the medium was set to 1.33, and the viscosity was set to 0.8872 cP, corresponding to water as the dispersant. Measurements were performed in triplicate after 120 min of equilibration at 25 °C and processed using Zetasizer Software 8.00. In addition to DLS measurements, particle morphology and physical size were analyzed using scanning electron microscopy (SEM). Fiji ImageJ software version ImageJ 1.54j (NIH, Bethesda, MD, USA) was used for image analysis.

#### 2.3.2. NPs Visualization

A Thermo Scientific Quattro scanning electron microscope (Waltham, MA, USA) was used to examine NP size, shape, and surface morphology. The particle suspension was deposited onto silicon substrates mounted on carbon adhesive tape. Samples were coated with a 5 nm gold layer using a Q150R ES Plus rotary-pumped coater (Quorum, Laughton, England) prior to imaging. SEM images were acquired under high vacuum at an accelerating voltage of 5 kV and a magnification of 80,000×.

A confocal laser scanning microscope (CLSM) (Leica TCS SP8 X, Leica, Wetzlar, Germany) equipped with a PLAN 100×/1.25 oil-immersion objective was used for visualization of Cy7-PLGA NPs. For imaging, the dye was excited at 671 nm, and emission was collected in the 720–800 nm spectral range (red channel).

#### 2.3.3. NPs Counting

Particle concentration was determined using an NP counter (NP Counter, NP VISION LLC, Vladivostok, Russia). The initial particle suspension was diluted 100,000-fold with ultrapure water to achieve a concentration within the dynamic range of the instrument. All measurements were performed in triplicate and processed using the manufacturer’s software, with results reported as mean ± standard deviation.

#### 2.3.4. Study of Encapsulated Dye Release

In vitro release testing of the Cy7-PLGA NPs was conducted using a sample-and-separate method in human blood plasma. Development of Level A in vitro–in vivo correlations for peptide-loaded PLGA microspheres [1,2,3,4,5,6,7,8,9,10,11,12,13]. For this purpose, the particle suspension was precipitated by centrifugation, resuspended in plasma, and incubated in an Eppendorf tube on a thermostatically controlled shaker at 37 °C in the dark, shaking at 300 rpm. At different time points (1, 24, 48, and 72 h), the samples were centrifuged at 13,200 rpm for 30 min. The supernatant was carefully collected and analyzed for dye content using UV spectroscopy at 760 nm. A calibration curve was obtained by adding known amounts of dye to blood plasma.

#### 2.3.5. Spectrofluorimetric Studies

A Tecan Infinite M Nano+ multifunctional microplate reader (Tecan Trading AG, Männedorf, Switzerland) was used for absorbance and fluorescence measurements of Cy7–PLGA NPs. All measurements were conducted at 25 °C. For analysis, 200 µL of each sample was transferred into individual wells of a UV-transparent 96-well plate (Corning Inc., Corning, NY, USA). Absorbance spectra were recorded in the 650–820 nm range with a 2 nm step size. Fluorescence spectra were obtained in top-reading mode with an excitation wavelength of λ_exc_ = 670 nm and emission collected in the 700–850 nm range, using 25 flashes per well and an integration time of 20 µs.

### 2.4. In Vitro Studies

#### 2.4.1. Cell Lines

HEK293 (human embryonic kidney), C2C12 (mouse myoblast), L929 (mouse fibroblast), and RAW 264.7 (mouse macrophage-like) cell lines were obtained from ATCC^®^. CT26 (murine colorectal carcinoma) and Renca (murine renal adenocarcinoma) cell lines were obtained from CLS^®^. Primary mMSCs (mouse mesenchymal stem cells) were obtained from the St. Petersburg State Chemical Pharmaceutical University cell collection. Primary white blood cells (WBCs) were isolated from human peripheral blood. RPMI (for Renca and WBCs) and DMEM (for all other cell lines), supplemented with 10% fetal bovine serum (FBS) and 1% penicillin–streptomycin (10,000 U/mL), were used for cell cultivation. All cells were maintained in a humidified incubator at 37 °C and 5% CO_2_, and subculturing was performed according to ATCC guidelines.

#### 2.4.2. Isolation of Primary White Blood Cells (WBCs)

Blood samples were anticoagulated with heparin. First, 5 mL of Ficoll was added to a 15 mL centrifuge tube at room temperature. Then, 5 mL of blood was carefully added along the tube wall and centrifuged at 400× *g* for 30 min. The WBCs fraction was then carefully transferred to a new 15 mL tube and washed three times with 15 mL of isotonic Ca^2+^/Mg^2+^-free DPBS (250× *g*, 10 min). Finally, 2 mL of RPMI supplemented with 10% FBS and 1% penicillin-streptomycin was added, and cells were counted using an automated cell counter.

#### 2.4.3. Cytotoxicity Assays

mMSCs, HEK 293, C2C12, L929, and RAW 264.7 were seeded in 96-well adhesive plates at a density of 5 × 10^3^ cells per well in 100 µL of growth medium and incubated overnight (~12 h) at 37 °C and 5% CO_2_ (five biological replicates, *n* = 5). The growth medium was then removed, and NPs suspensions were added in 200 µL of fresh growth medium at ratios of 1, 2, 4, 6, and 8 × 10^6^ NPs per cell. Cells cultured without NPs served as controls. After 48 h of incubation, the medium was removed and replaced with 100 µL of fresh growth medium containing 10% resazurin stock solution (0.5 mg/mL in DPBS), followed by incubation at 37 °C for 3 h. Cell viability was assessed by fluorescence spectrophotometry (excitation/emission: 560/590 nm) using a Tecan Infinite M Nano+ multifunctional microplate reader (Tecan Trading AG, Männedorf, Switzerland).

WBCs were seeded in 96-well plates at a density of 5 × 10^4^ cells per well in 100 µL of growth medium (five biological replicates, *n* = 5) and incubated overnight (~12 h) at 37 °C and 5% CO_2_. NPs were added at ratios of 1, 2, 4, 6, 8, 10, 15, and 20 × 10^4^ NPs per cell in 100 µL of growth medium. Cells without NPs served as controls. After 48 h, 100 µL of fresh growth medium containing 30% resazurin stock solution (0.5 mg/mL in DPBS) was added to each well and incubated at 37 °C for 3 h. Viability was assessed as described above for adherent cell cultures.

Endotoxin content of the NPs preparation was not assessed in this study.

#### 2.4.4. Hemolysis Assay

Blood samples (2 mL) anticoagulated with heparin were centrifuged at 400× *g* for 5 min. Plasma was removed, and red blood cells (RBCs) were washed three times with 2 mL of isotonic Ca^2+^/Mg^2+^-free DPBS by centrifugation under identical conditions. The RBC suspension was then diluted 1:100 in DPBS to obtain a 1% erythrocyte suspension. Aliquots of 100 µL of the RBC suspension (~5 × 10^6^ cells) were mixed with 100 µL of NP suspensions (2.5, 5, 10, 15, and 20 × 10^10^ particles per 100 µL DPBS) in 2 mL centrifuge tubes (three replicates, *n* = 3). Thus, particle-to-cell ratios of 0.5, 1, 2, 3, and 4 × 10^4^ particles per cell were obtained. Triton X-100 (10%) was used as a positive control, and DPBS served as a negative control. Samples were incubated at 37 °C for 60 min and centrifuged at 400× *g* for 5 min. Finally, 100 µL of the supernatant was transferred to a 96-well plate, and absorbance was measured at 405 nm using a microplate reader.

### 2.5. In Vivo and Ex Vivo Studies

#### 2.5.1. Animals

All animal procedures were conducted in accordance with the ethical guidelines of Ogarev Mordovia State University (protocol No. 139 by 30 April 2025) and the International Guiding Principles for Biomedical Research Involving Animals (Geneva Convention, 1985). Experiments were carried out on white BALB/c mice aged 6–8 weeks and weighing 20–25 g under general anesthesia. Anesthesia was induced using a Zoletil-based mixture (40 mg/kg, 50 µL; Virbac SA, Carros, France) and 2% Rometar (10 mg/kg, 10 µL; Spofa, Jičín, Czech Republic), administered intraperitoneally. Each experimental group consisted of 5–6 animals, except for blood flow assessment, which involved 5 mice per group. At the end of the experiments, animals were euthanized by anesthetic overdose.

#### 2.5.2. Cancer Models

In vivo studies of NP biodistribution following intra-arterial administration were performed using two murine tumor models. For femoral artery targeting, 1 × 10^6^ CT26 (murine colorectal carcinoma) cells were implanted subcutaneously into the left hind paw in 10 µL of RPMI. An orthotopic renal cancer model was used for renal artery targeting. Tumors were established by subcapsular injection of 2.5 × 10^5^ Renca (murine renal cortical adenocarcinoma) cells into the left kidney in 10 µL of RPMI [39]. Catheterization and subsequent NP biodistribution studies were initiated 7 days after cell implantation.

#### 2.5.3. Carotid Artery Catheterization

A detailed description of the carotid artery catheterization procedure is provided in the Supplementary Materials (Information S1).

Cy7-labeled NPs were administered via a polyethylene catheter (PE-10) (Scientific Commodities Inc., Lake Havasu City, AZ, USA) equipped with polyurethane tubing (40 mm, 32 ga/0.8 Fr; Instech, Rotterdam, The Netherlands). A 20 μL suspension containing 2.5 × 10^10^ NPs (adjusted for a 5 μL dead volume) was injected over 10–15 s. Following injection, the catheter was removed, the puncture site was sealed with tissue adhesive, blood flow was restored, and the incision was closed with sutures.

#### 2.5.4. Femoral and Renal Artery Catheterization

A detailed description of femoral and renal artery catheterization procedures is provided in our previously published protocol [29] and in the Supplementary Materials (Information S2).

A saline-filled PE-10 catheter with polyurethane tubing (40 mm, 32 ga/0.8 Fr; Instech) was inserted into the left renal artery via the right femoral artery and abdominal aorta. Partial renal blood flow was maintained because the renal artery diameter slightly exceeded that of the catheter. A 20 μL suspension containing 2.5 × 10^10^ Cy7-labeled NPs (adjusted for a 5 μL dead volume) was injected into the left renal artery over 10–15 s. To compensate for the dead volume, an additional 5 µL of suspension was administered during the injection. During injection, the renal artery was gently compressed around the catheter to prevent reflux into the aorta. After 1 min, the forceps were released, the catheter was withdrawn, and the femoral artery was ligated. Muscle and skin were sutured to complete the procedure.

#### 2.5.5. Blood Flow Measurement

Blood flow dynamics following NP administration, an important indicator of procedural safety, were assessed in vivo using a custom-built laser speckle contrast imaging (LSCI) system for real-time, non-contact monitoring, as previously described [26]. Due to the limited penetration depth of laser radiation and strong tissue scattering, measurements required direct optical access to the vessel, organ, or tissue of interest. Therefore, a surgical incision was created to provide an optical window.

Blood flow in the left carotid artery was measured before and 15 min after catheterization, NP injection, and sealing of the catheter insertion site. Blood flow in the left kidney was assessed before and after renal artery catheterization, NP administration, and catheter removal. Left femoral artery blood flow was evaluated before and immediately after right femoral artery catheterization, NP injection, and catheter withdrawal.

Data acquisition commenced after systemic hemodynamic stabilization and continued for 2 min. To improve image quality, 25 consecutive frames were averaged, and blood flow maps were smoothed using a Gaussian filter (σ = 7) to suppress high-frequency noise. A 200 × 200 pixel region of interest (ROI) was analyzed for each dataset, and mean ± standard deviation values were extracted. Data processing was performed using NumPy v1.16.5 and SciPy v1.3.1 libraries in Python 3.6.

#### 2.5.6. Biodistribution Analysis

Biodistribution of fluorescence in murine organs following NP administration was evaluated using the IVIS SpectrumCT In Vivo Imaging System (PerkinElmer, Waltham, MA, USA) with excitation/emission settings of 745/800 nm. Data were analyzed using Living Image software v4.7.3. The administered NPs containing Cy7 served as photon emission sources upon excitation. Due to minimal tissue autofluorescence in the near-infrared range, NP-derived fluorescence was readily distinguished from background signals.

Fluorescence localization efficiency was assessed both in vivo and ex vivo. Two experimental groups received 2.5 × 10^10^ NPs via carotid, renal, or femoral arteries using a constant injection volume of 20 µL, accounting for a 5 µL catheter dead volume. In vivo imaging was performed at 15 min, 1 h, 24 h, 48 h, and 72 h post-injection. Animals were subsequently euthanized, and organs were collected for ex vivo imaging at 15 min, 24 h, and 72 h. Results were compared with NP biodistribution following tail vein injection at identical dose and volume (in vivo: 15 min, 1 h, 24 h, 48 h, 72 h; ex vivo: 15 min).

Quantitative fluorescence analysis was performed by determining the Total Radiant Efficiency within ROIs defined by anatomical organ boundaries. Total Radiant Efficiency, the gold-standard metric for fluorescence imaging, reflects cumulative photon flux per second from the ROI normalized to excitation intensity. For each organ, values were background-corrected using signals from the control group. Organ weights were not recorded and were not included in the analysis.

#### 2.5.7. Cryosections Preparation

Animals subjected to carotid artery NP administration received 200 µL of 5% Evans Blue dye in saline intravenously immediately and 24 h post-procedure. After a 15 min circulation period, animals were euthanized by anesthetic overdose. Thus, brain tissues were analyzed 15 min and 24 h after arterial NP administration. In this experiment, Cy7 was replaced with Cy3.5 in NPs to enable spectral separation from Evans Blue-stained vessels.

Animals receiving Cy7-labeled NPs via renal or femoral artery catheterization were injected intravenously with 200 µL of 1% fluorescently labeled 500 kDa FITC-dextran 13 min and 24 h post-procedure for vessel visualization. After a 2 min circulation period, euthanasia was performed by anesthetic overdose. Target tissues (kidney, muscle, tumors) were analyzed 15 min and 24 h after NP administration.

Following euthanasia, tissues were fixed in 10% neutral buffered formalin for 24–48 h, then transferred to DPBS, which was replaced three times over 24 h. Samples were cryoprotected in sucrose solutions (10% for 2 h, 20% for 2 h, and 30% overnight) and frozen at −20 °C.

Cryosections (70 µm) were prepared using a Leica CM1950 cryostat (Leica Microsystems, Germany). Chamber temperature was −20 °C, and sample temperature was −10 °C. Sections were stained with 0.1% DAPI prior to imaging.

#### 2.5.8. Confocal Microscopy of Cryosections

Cryosections were examined using a Leica TCS SP8 X microscope (Leica, Germany) equipped with a 20×/0.70 NA objective. CLSM enabled detailed visualization and spatial distribution analysis of NP aggregates.

For kidney, muscle, and tumor imaging, fluorescence channels were configured as follows: DAPI (excitation 405 nm, emission 420–475 nm, nuclei), FITC–dextran (excitation 488 nm, emission 520–580 nm, vessels), and Cy7-labeled NPs (excitation 671 nm, emission 720–800 nm, red channel).

For brain imaging, fluorescence detection included DAPI (405 nm excitation, 420–475 nm emission), Evans Blue (excitation 561 nm, emission 650–750 nm, red channel), and Cy3.5-labeled NPs (excitation 561 nm, emission 575–630 nm, yellow channel).

#### 2.5.9. Histological Analysis

Histological evaluation of target organs was performed 120 h (5 days) after arterial NP administration. Tissues were fixed in 10% neutral buffered formalin, dehydrated through a graded isopropanol series, embedded in paraffin, sectioned (5 µm), and stained with hematoxylin and eosin (H&E). Morphological assessment was conducted using an Olympus digital imaging system.

### 2.6. Statistical Analysis

All quantitative in vitro and ex vivo data are presented as mean ± standard deviation (SD). Statistical analyses were performed using GraphPad Prism v8.2.1. An unpaired *t*-test with Welch’s correction was applied to assess differences between experimental groups. Statistical significance was defined as follows: ns, *p* > 0.05; *, *p* < 0.05; **, *p* < 0.01.

## 3. Results

### 3.1. NPs Synthesis and Characterization

The hydrophobic fluorescent dye Cy7 was selected as a model substance for incorporation into PLGA particles to evaluate their biological distribution in living organisms. Cy7 is a near-infrared fluorescent dye with a high quantum yield that is readily visualized in the first tissue transparency window and is well suited for assessing biodistribution in mice [40,41,42,43].

**Figure 2:**
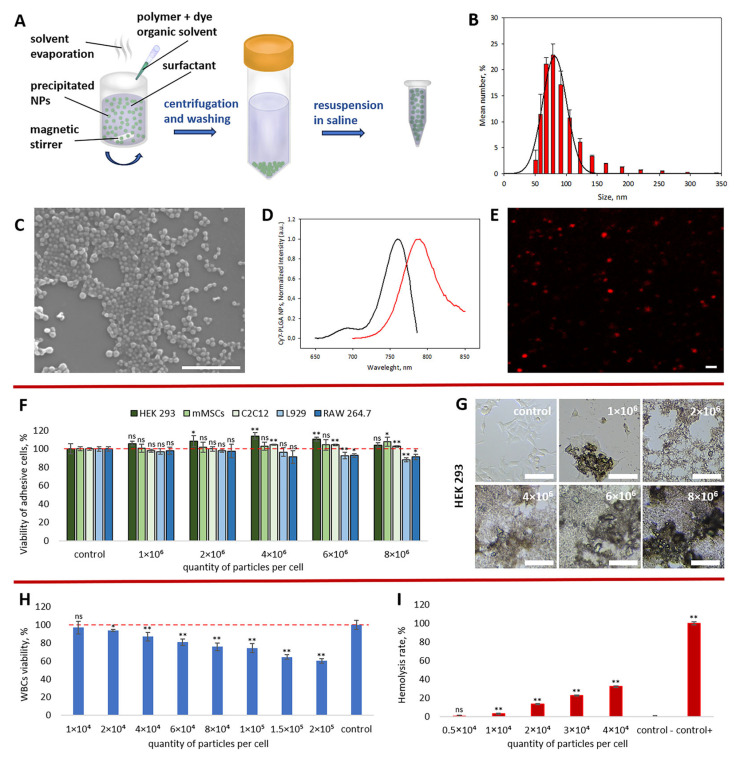
Schematic illustration of Cy7-PLGA NP preparation (**A**). NP size distributions measured by DLS and presented as mean ± standard deviation, with Gaussian curve fitting (**B**). Representative SEM image of the NPs (scale bar: 1 µm; magnification: ×80,000) (**C**). Normalized absorption (black line) and fluorescence spectra (red line) of Cy7 encapsulated in PLGA NPs (**D**) and CLSM image of Cy7–PLGA NP aggregates (scale bar: 1 µm) (**E**). Cytotoxicity of NPs toward HEK 293, mMSCs, C2C12, L929, and RAW 264.7 cell lines 48 h after exposure at ratios of 1, 2, 4, 6, and 8 × 10^6^ NPs per cell (*n* = 5) (**F**). Optical microscopy images of HEK 293 cells 48 h after NP exposure under the same conditions (scale bar: 100 µm) (**G**). Hemocompatibility of NPs toward WBCs (*n* = 5) (**H**) and RBCs (*n* = 3) (**I**), 48 h and 1 h after exposure, respectively. All in vitro data are presented as mean ± standard deviation (SD). Statistical analysis was performed relative to the control group for viability and the negative control for hemolysis (without NP addition) using an unpaired *t*-test with Welch’s correction. Statistical significance is defined as follows: ns, *p* > 0.05; *, *p* < 0.05; **, *p* < 0.01.

PLGA NPs were prepared by the nanoprecipitation technique, a solvent displacement method similar to that employed by Fessi et al., with minor modifications [38]. Briefly, predetermined amounts of PLGA and Cy7 were dissolved in acetone and injected into an appropriate volume of an aqueous solution containing Tween–80 as a surfactant (Figure 2A). The temperature of the dispersion medium was maintained at the boiling point of the solvent, which facilitated rapid solvent diffusion into the aqueous phase, increased the particle formation rate, and accelerated solvent evaporation. The mixture rapidly became slightly opalescent due to the formation of polymer NPs. Cy7, a hydrophobic dye, was incorporated into the polymer matrix by physical sorption [44]. In this regard, four washing steps were performed to remove excess stabilizer and dye that was not included in the polymer particles.

The sizes of the Cy7-PLGA NPs were measured by dynamic light scattering. The PLGA NPs were suspended in water and characterized using a Zetasizer Nano ZS (Malvern Instruments, Malvern, UK). The average particle size determined by number-weighted distribution was 92.9 ± 37.7 nm (Figure 2B). The hydrodynamic diameter was determined by DLS Z-average value of 182.3 ± 1.9 nm, with a polydispersity index (PDI) of 0.352. The difference in these values can be attributed both to the formation of a small number of aggregates as a result of extensive washing of the particles and to interactions between individual Cy7-PLGA NPs arising from their hydrophobic nature. T. Phenrat et al. previously reported an increase in the average particle size in the intensity-weighted distribution compared to the number-weighted distribution for interacting magnetic particles in an aqueous medium [45]. We suggest that a similar effect may occur in the case of PLGA nanoparticles loaded with hydrophobic compounds. A comparison of intensity- and number-weighted size distributions is shown in Figure S1 (Supplementary Materials). To further characterize the physical dimensions of the particles, images obtained using scanning electron microscopy (SEM) were analyzed. The SEM study showed that Cy7-PLGA NPs were spherical with a smooth surface, with an average diameter of 89.5 ± 26.3 nm (PDI = 0.09) and were monodisperse across different fields of view (Figure 2C). A summary of Cy7-PLGA NP size distribution obtained from DLS and SEM analyses is provided in Table S1 (Supplementary Materials). The particle size values obtained under the described conditions were consistent with data from systematic studies investigating the effect of precipitation parameters on NP size distribution [46]. The typical value of ζ-potential of the particles was −24.4 ± 5.9 mV, which is a common value for negatively charged PLGA particles, as reported in numerous studies [1,47]. For subsequent in vivo experiments, the particles were transferred to physiological saline, and a suspension with a known particle concentration (particles/mL) was used.

To confirm that the PLGA NPs were loaded with fluorescent dye, the absorption and fluorescence spectra of the particle suspension were recorded (Figure 2D). The resulting Cy7-PLGA particles exhibited intense fluorescence in the near-infrared spectral range. Figure 2E shows confocal laser scanning microscopy (CLSM) images of the particles, where the brightest signals correspond to aggregates, due to the limited optical resolution of the microscope.

An initial burst release of Cy7 (~53%) was observed during the first hour of incubation, likely associated with desorption of dye localized on the particle surface, as reported by several authors [48]. In addition, Tween 80, used during particle production, may accelerate the transfer of dye into the surrounding medium [49]. Following the initial release, the release profile indicates a sustained release of up to 66% over the next three days, likely due to diffusion of physically adsorbed dye from the PLGA polymer matrix (Figure S2).

### 3.2. Cytotoxicity and Hemocompatibility Studies

Detailed studies of the cytotoxicity and hemocompatibility of PLGA-Cy7 NPs in cell cultures were conducted using both adhesive and suspension cell models. HEK 293 (human embryonic kidney cells), C2C12 (mouse myoblasts), L929 (mouse fibroblasts), and RAW 264.7 (mouse macrophage-like cells) adhesive cell lines were chosen as models of normal cells present in the brain, kidney, and hind paw muscle. Incubation for 48 h with NPs at a ratio of 6 × 10^6^ NPs per cell did not lead to significant changes in the viability of HEK 293, mMSCs, or C2C12 cells (Figure 2F). A modest but statistically significant decrease in viability under the same conditions was observed for L929 and RAW 264.7, with values of 92.6 ± 3.4% and 92.8 ± 2.1%, respectively. At such high concentrations (4, 6, and 8 × 10^6^ NPs per cell), the cells were covered with a dense layer of NPs, as illustrated by the HEK 293 cell example (Figure 2G).

Hemocompatibility studies revealed toxic effects of the NPs on primary white blood cell (WBC) cultures, but only at relatively high concentrations (Figure 2H). Thus, a statistically significant decrease in WBC viability 48 h after incubation was observed starting from a ratio of 2 × 10^4^ NPs per cell, with a viability of 93.7 ± 1.1%. At the same time, IC_50_ was not reached even at a ratio of 2 × 10^5^ NPs per cell, where leukocyte survival remained 60.1 ± 2.6%. Statistically significant hemolysis of red blood cells (RBCs) was observed starting from a concentration of 2.5 × 10^10^ NPs per mL, corresponding to a ratio of 1 × 10^4^ NPs per cell (Figure 2I). At this ratio, the hemolysis level was 2.9 ± 0.6%. Increasing the ratio to 4 × 10^4^ NPs per cell resulted in a hemolysis level of 32.1 ± 0.7%.

Overall, the data indicate low cytotoxicity and good hemocompatibility of the synthesized NPs. It should be noted that the incorporation of additional components (therapeutic agents or biologically active molecules) into NPs may increase their toxicity. Therefore, upon replacement of the model dye with a drug, cytotoxicity and hemocompatibility must be additionally evaluated.

### 3.3. NPs Targeting in Brain and Salivary Glands Using Carotid Artery Injection

**Figure A1:**
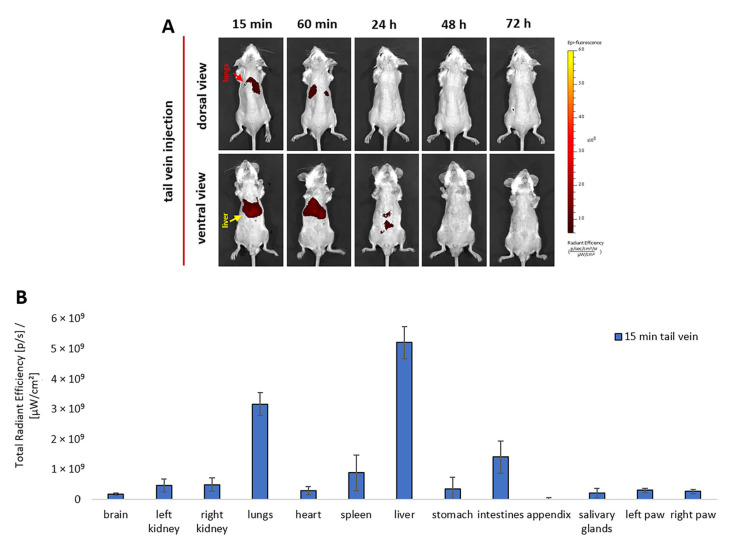
Dynamics of fluorescence biodistribution during 72 h after tail vein injection of Cy7-labeled NPs in vivo (**A**) and ex vivo 15 min post-injection (**B**).

In the first stage of the in vivo studies, PLGA-Cy7 NPs were administered via the left carotid artery to achieve targeting and retention in the cerebral vasculature using the first-pass effect. It should be noted that intravenous administration produced the expected biodistribution pattern for this type of NPs (Figure A1), consistent with previously reported data [6].

**Figure 3:**
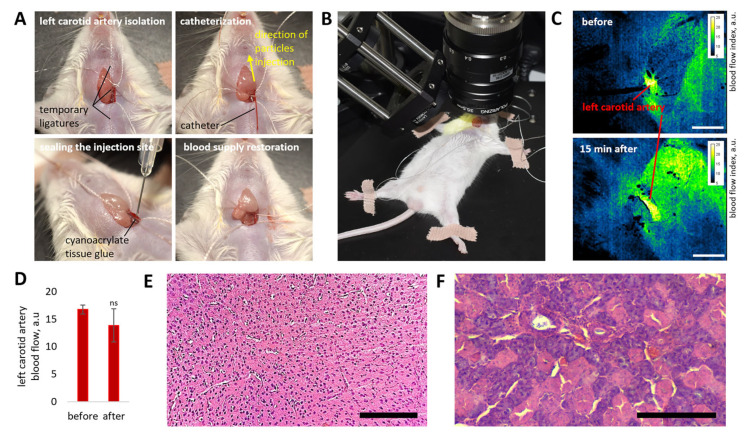
Photographic documentation of the main stages of carotid artery catheterization and sealing of the catheter insertion site (**A**). Blood flow measurements in the left carotid artery before and after catheterization, injection of 2.5 × 10^10^ NPs, and sealing, obtained using a custom laser speckle contrast imaging (LSCI) setup (**B**). Representative LSCI images of the carotid artery before and after NP injection (scale bar: 1 mm) (**C**). Average carotid artery blood flow values (*n* = 4) before and 15 min after NP injection and sealing (**D**). Data are presented as mean ± SD. Statistical analysis was performed relative to baseline (before NP administration) using an unpaired *t*-test with Welch’s correction (ns, *p* > 0.05). Representative histological images of brain tissue (**E**) and salivary gland (**F**) from the target brain hemisphere 5 days after carotid artery NP injection, demonstrating normal tissue morphology. Section thickness: 5 µm; scale bar: 200 µm.

Initially, a method for sealing the carotid artery and restoring normal blood supply after catheterization was developed to ensure effective and safe delivery of NPs to brain tissue. After catheter implantation and NPs injection, the catheter was carefully removed (Figure 3A). The upper ligature (located closer to the head) was then briefly loosened (for less than a second) to ensure that the punctured region of the artery was filled with blood.

Only after this step was the catheter implantation site sealed with medical-grade cyanoacrylate adhesive for tissues and vessels. Sealing the blood-filled carotid artery allowed us to preserve blood flow after all manipulations, which was confirmed by data obtained using laser speckle contrast imaging (LSCI) setup (Figure 3B,C). According to LSCI analysis, no significant differences in blood flow parameters of the left carotid artery were observed before manipulation and 15 min afterward (Figure 3D). Histological analysis of brain tissue and salivary glands performed 5 days after the procedure also did not reveal pathological changes; the brain tissue retained its normal morphological structure, and no neuronal necrosis was observed (Figure 3E,F).

**Figure A2:**
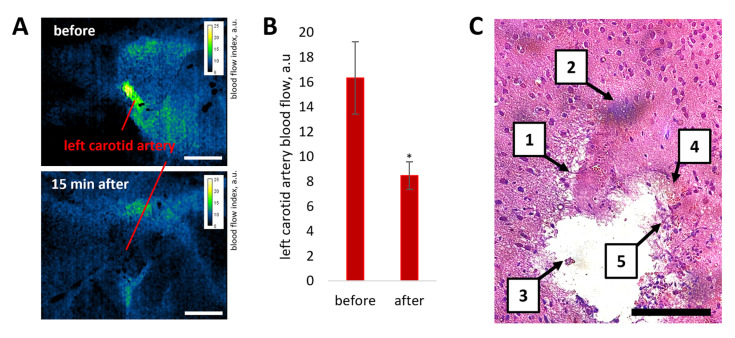
Representative LSCI images of the carotid artery before and after incorrect NP injection, resulting in carotid artery occlusion, obtained using the laser speckle contrast imaging (LSCI) setup (scale bar: 1 mm) (**A**). Average carotid artery blood flow values (*n* = 3) before and 15 min after NP injection with incorrect sealing of the catheter insertion site, leading to partial or complete blood flow obstruction (**B**). Blood flow data are presented as mean ± standard deviation (SD). Statistical analysis was performed relative to baseline (before NP administration) using an unpaired *t*-test with Welch’s correction (*, *p* < 0.05). Representative histological image of brain tissue from the target hemisphere 5 days after incorrect carotid artery NP injection, demonstrating pathological changes, including: (1) neuronal death; (2) hemosiderosis (blood impregnation); (3) cavities filled with cerebrospinal fluid, edema, and tissue disintegration; (4) microhemorrhages; and (5) leukocyte infiltration (**C**). Section thickness: 5 µm; scale bar: 200 µm.

It is important to note that if the carotid artery was not sealed correctly (i.e., not filled with blood), blood flow was not restored fully or partially (Figure A2A,B). In this case, histological analysis revealed pathological changes in brain tissue 5 days after manipulation, including sites of neuronal death, foci of hemosiderosis (blood impregnation), cavities filled with cerebrospinal fluid, edema, tissue disintegration, microhemorrhages, and leukocyte infiltration (Figure A2C). Overall, these data emphasize the importance of maintaining normal blood flow in the carotid artery and monitoring it following intra-arterial NP administration to ensure experimental correctness.

**Figure 4:**
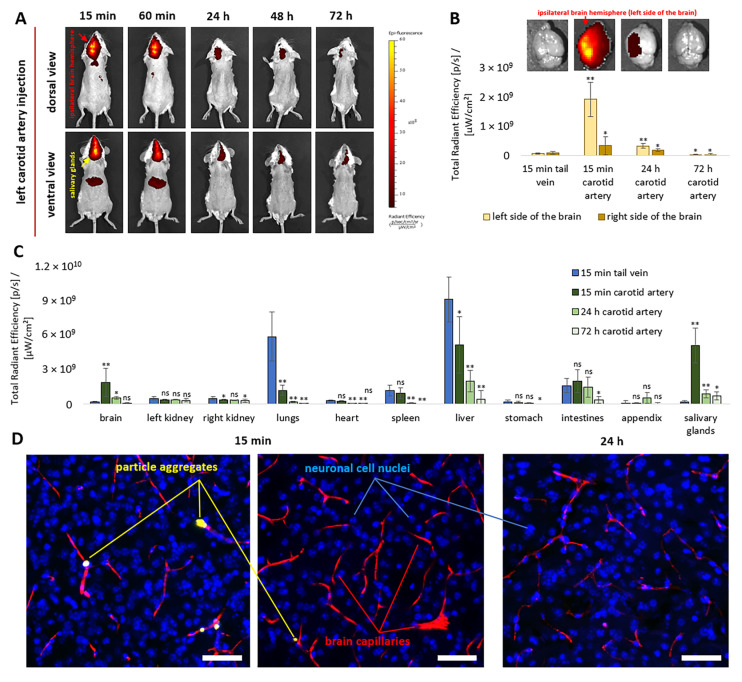
Dynamics of fluorescence biodistribution during 72 h following left carotid artery injection of 2.5 × 10^10^ Cy7-labeled NPs in vivo (**A**) and ex vivo in the brain (**B**) and other organs (**C**), compared with tail vein injection (*n* = 6 per each group). Representative confocal microscopy images of brain tissue 15 min and 24 h after carotid artery NP injection. Blue indicates cell nuclei (DAPI), red indicates brain capillaries (Evans Blue), and yellow indicates NP aggregates (Cy3.5-labeled) (**D**). Cryosection thickness: 70 µm; scale bar: 50 µm. All ex vivo data are presented as mean ± SD. Statistical analysis was performed relative to the control group (15 min tail vein injection) using an unpaired *t*-test with Welch’s correction. Statistical significance: ns, *p* > 0.05; *, *p* < 0.05; **, *p* < 0.01.

In vivo analysis of NP biodistribution dynamics following catheterization of the left carotid artery confirmed the effectiveness of the proposed strategy (Figure 4A). A bright fluorescent signal was predominantly observed in the left hemisphere of the brain and in the salivary glands, mainly in the submandibular glands. Moreover, the fluorescence intensity in these regions exceeded that observed in the liver and decreased over time.

A more detailed ex vivo analysis confirmed significant differences between the two routes of NP administration (left carotid artery versus tail vein) 15 min after injection (Figure 4B). Fluorescent signal intensities in the left and right hemispheres of the brain were 31.5-fold and 3.4-fold higher, respectively, than those measured after intravenous administration. After 24 h, these differences decreased to 5.4-fold and 1.8-fold, respectively. A similar trend was observed for the salivary glands, where fluorescence intensity was 28.3-fold, 4.9-fold, and 3.8-fold higher at 15 min, 24 h, and 72 h, respectively, compared with intravenous administration (Figure 4C). In general, the injection of NPs through the left artery radically changed the biodistribution of the fluorescent signal from the encapsulated dye Cy7 at 15 min, significantly reducing it in the liver (1.8-fold) and lungs (1.6-fold) and increasing it in the brain (10-fold), salivary glands (28.3-fold), and intestines (1.2-fold). The ROI fluorescence values presented in Figure 4B,C were not normalized to organ weight but were normalized to autofluorescence variability.

Analysis of confocal images of cryosections allowed visualization of NPs only as small aggregates (2–14 µm) within individual brain vessels (Figure 4D). Nevertheless, these structures were no longer detected in brain vessels after 24 h. The absence of irreversible vessel occlusion by NPs is an important criterion for the safety of their use in brain drug delivery. It is important to note that no foci of Evans’ blue dye extravasation from cerebral vessels were detected, indicating the absence of ischemic or hemorrhagic damage, as well as preservation of blood–brain barrier (BBB) integrity. However, the absence of clear Evans’ blue leakage permitted only qualitative, rather than quantitative, assessment of potential damage, likely due to the limited spatial resolution of the technique. Importantly, these findings were fully consistent with the histological analysis shown in Figure 3E.

### 3.4. NPs Targeting in Kidney with Tumor Using Renal Artery Injection

**Figure 5:**
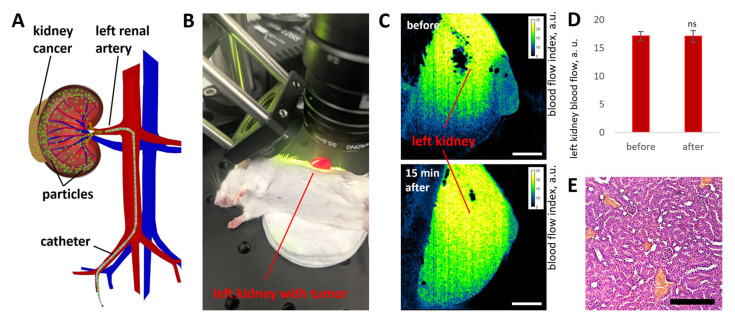
Schematic illustration of renal artery catheterization and intra-arterial NP targeting in mice with an orthotopic kidney cancer model (**A**). Blood flow measurements at the surface of the target kidney before and 15 min after injection of 2.5 × 10^10^ NPs using the LSCI setup (**B**). Representative LSCI images obtained before and after NP injection (scale bar: 1 mm) (**C**). Average kidney blood flow values (*n* = 5) before and 15 min after renal artery NP injection (**D**). Data are presented as mean ± SD; statistical analysis was performed relative to baseline using an unpaired *t*-test with Welch’s correction (ns, *p* > 0.05). Representative histological image of kidney tissue from healthy mice 5 days after renal artery NP injection, showing normal morphology (section thickness: 5 µm; scale bar: 200 µm) (**E**).

In the second stage of the in vivo studies, the efficiency of NP delivery to the kidneys of mice bearing an orthotopic renal cell carcinoma model induced by subcapsular implantation of 2.5 × 10^5^ Renca cells was assessed (Figure 5A). In this case, the catheter was implanted through the femoral artery into the aorta and subsequently advanced into the left renal artery without compromising arterial integrity. As a result, renal artery blood supply was fully preserved following all interventions and catheter removal.

**Figure A3:**
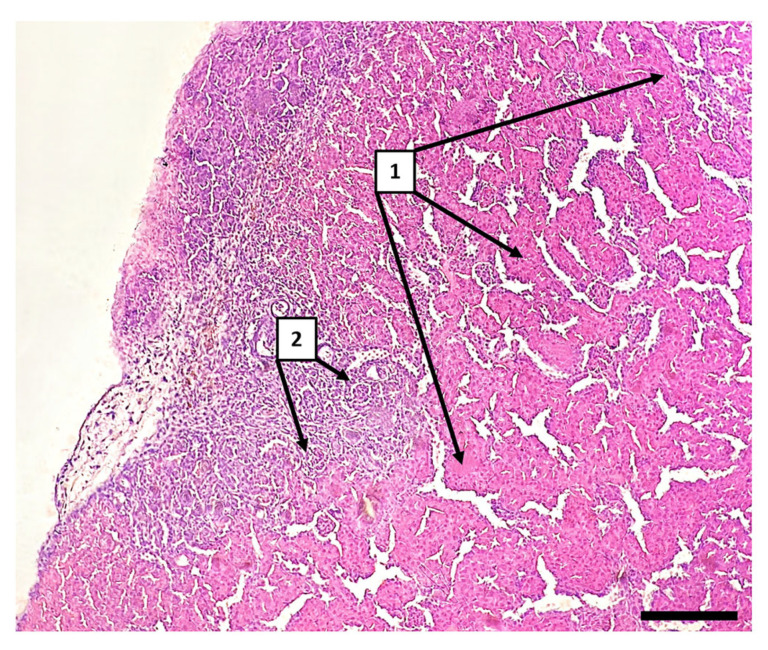
Representative histological image of kidney tissue with carcinoma 9 days after subcapsular implantation of 2.5 × 10^5^ Renca cells, demonstrating pathological alterations, including: (1) occluded renal tubules with dystrophic epithelium; (2) absence of Bowman’s capsule due to compression by edema, likely caused by inflammation in regions of active invasive tumor growth. Deformational changes were localized and observed only in areas of Renca cell invasion. Section thickness: 5 µm; scale bar: 200 µm.

Assessment of microcirculation in the vessels of the target kidney before and 15 min after all manipulations using the LSCI setup (Figure 5B,C) did not reveal any blood flow disturbances associated with NP delivery (Figure 5D). Histological analysis of the target organ also revealed no structural abnormalities in healthy kidneys 5 days after arterial NP delivery (Figure 5D). Histological analysis was performed in healthy animals because tumor progression in subsequent days leads to pronounced pathological alterations in kidney tissue (Figure A3), which could otherwise be misinterpreted as adverse effects of arterially administered NPs.

**Figure 6:**
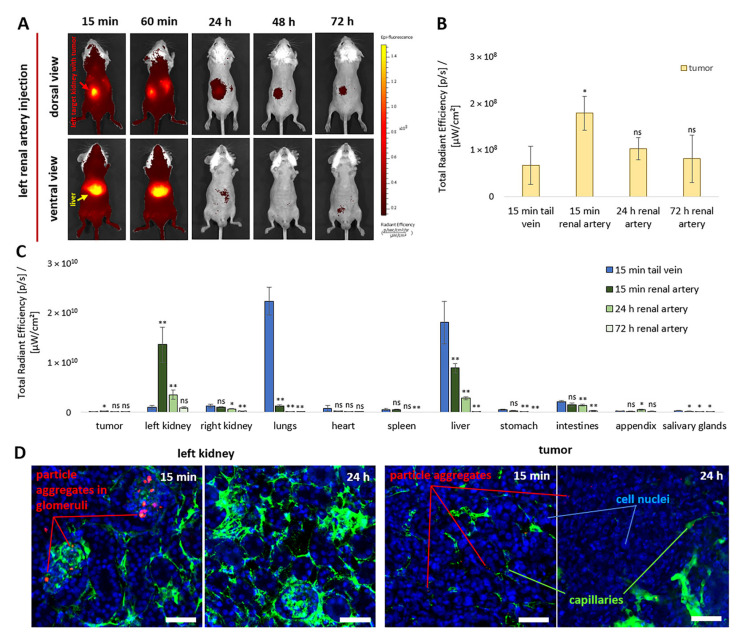
Fluorescence biodistribution dynamics during 72 h following left renal artery injection of 2.5 × 10^10^ Cy7-labeled NPs in vivo (**A**) and ex vivo in the tumor (**B**) and other organs (**C**), compared with tail vein injection (*n* = 5 per group). Representative confocal microscopy images of target kidney and tumor tissues 15 min and 24 h after NP injection. Blue indicates nuclei (DAPI), green indicates vessels (FITC–dextran, 500 kDa), and red indicates NP aggregates (Cy7) (**D**). Cryosection thickness: 70 µm; scale bar: 50 µm. All ex vivo data are presented as mean ± SD. Statistical analysis was performed relative to the control group (15 min tail vein injection) using an unpaired *t*-test with Welch’s correction. Statistical significance: ns, *p* > 0.05; *, *p* < 0.05; **, *p* < 0.01.

Targeting NPs via the renal artery resulted in a marked enhancement of fluorescence in the target kidney region (Figure 6A). Although the fluorescent signal from the liver was also initially strong, it decreased more rapidly during the subsequent observation period. Renal arterial delivery resulted in at least a 2.7-fold increase in NP accumulation in the tumor compared with intravenous administration at 15 min post-injection (Figure 6B). Moreover, no differences in fluorescence intensity were observed in tumor tissue 24 h after intra-arterial administration and 15 min after intravenous administration.

The efficiency of NP accumulation in the target kidney was significantly higher (Figure 6C). At 15 min following arterial administration, fluorescence intensity was at least 13.7-fold higher than that observed after intravenous injection. After 24 h, fluorescence intensity decreased but remained 3.5-fold higher, whereas after 72 h the differences were no longer detectable. Overall, the biodistribution profiles of NPs changed markedly following renal arterial administration compared with intravenous injection (Figure 6C). Renal arterial delivery not only increased NP accumulation in the kidney and tumor but also substantially reduced off-target exposure of the lungs, liver, and salivary glands at 15 min post-administration by at least 18.3-fold, 2-fold, and 1.8-fold, respectively.

**Figure A4:**
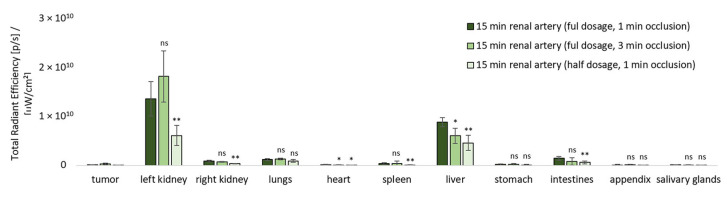
Ex vivo fluorescence biodistribution 15 min after left renal artery injection of full (2.5 × 10^10^) and half (1.25 × 10^10^) doses of Cy7-labeled NPs. For the full dose, data are shown following temporary renal artery occlusion (1 or 3 min) after intra-arterial NP administration. Ex vivo data are presented as mean ± standard deviation (SD, *n* = 5). Statistical analysis was performed relative to the control group (15 min renal artery injection, full dose, 1 min occlusion) using an unpaired *t*-test with Welch’s correction. Statistical significance: ns, *p* > 0.05; *, *p* < 0.05; **, *p* < 0.01.

Notably, increasing the duration of temporary renal artery occlusion after NP administration from 1 to 3 min did not result in a significant increase in fluorescence intensity in the target kidney or tumor (Figure A4), indicating that prolonged blood-flow interruption is unnecessary for improving delivery efficiency. In addition, a two-fold reduction in NP dose (1.25 × 10^10^ vs. 2.5 × 10^10^ NPs in 20 μL saline) led to a proportional decrease in fluorescence intensity across all organs. This behavior agreed upon from that observed for polyelectrolyte microcapsules (3.7 μm) [25], for which dose-dependent increases in renal accumulation were previously reported.

Confocal microscopic analysis of cryosections revealed the accumulation of a large number of NP aggregates 4–11 μm in size, mainly in the glomerular capillary network of the kidneys 15 min after injection (Figure 6D). In contrast, aggregates detected in tumor tissue were significantly smaller (1–5 μm) and considerably less frequent. At the same time, aggregates were almost undetectable in both renal and tumor tissues at 24 h. The absence of irreversible occlusion of the target renal vessels, together with the ability of FITC-dextran to penetrate the glomerular capillaries after NP aggregate accumulation, indicates the preservation of normal renal blood circulation. These data are critical for confirming the safety of the developed approach.

### 3.5. NPs Targeting in Hind Paw with Tumor Using Femoral Artery Injection

**Figure 7:**
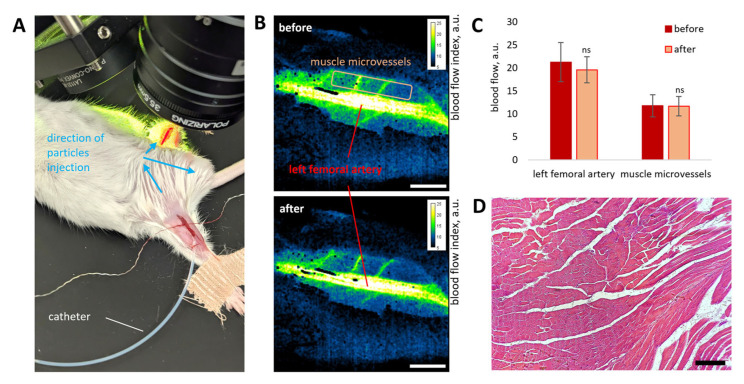
Blood flow measurements in the left (target) hind paw bearing a CT26 tumor (7 days) and left femoral artery before and immediately after right femoral artery NP injection using LSCI. The catheter was inserted into the right femoral artery to a depth of 2.5 cm. Blue arrows indicate NP flow direction (ventral tail artery, left femoral artery, and tumor-bearing hind paw) during injection (**A**). Representative LSCI images of the left femoral artery and hind paw muscle microvasculature before and after NP injection (scale bar: 1 mm) (**B**). Average blood flow values (*n* = 5) in the target hind paw muscle and left femoral artery before and immediately after NP injection (**C**). Data are presented as mean ±SD; statistical analysis was performed relative to baseline using an unpaired *t*-test with Welch’s correction (ns, *p* > 0.05). Representative histological image of hind paw muscle from healthy mice 5 days after femoral artery NP injection, demonstrating normal tissue morphology (section thickness: 5 µm; scale bar: 200 µm) (**D**).

In the final stage of the study, we targeted the left hind paw bearing a 7-day-old tumor induced by subcutaneous injection of 1 × 10^6^ CT26 cells (murine colorectal carcinoma). For this purpose, the catheter was implanted into the right femoral artery approximately 2 cm proximal to the bifurcation of the abdominal artery into the femoral arteries and ventral tail artery (Figure 7A). Analysis of blood flow in the left femoral artery and adjacent muscle microvessels (Figure 7B) did not reveal significant differences before and immediately after NP injection (Figure 7C). Standard histological analysis performed 5 days after NP injection via the femoral artery revealed no obvious pathological changes in tissue structure (Figure 7D). Nevertheless, the possibility of acute inflammatory reactions or tissue damage at earlier time points cannot be excluded, as such effects may have been mitigated by day 5 for this type of carrier. Furthermore, changes in the carrier type, the chemical composition of its components, or the inclusion of therapeutic agents may increase the risk of tissue damage at both early and late stages after administration. These factors should therefore be carefully considered and systematically evaluated in both experimental and translational studies.

**Figure 8:**
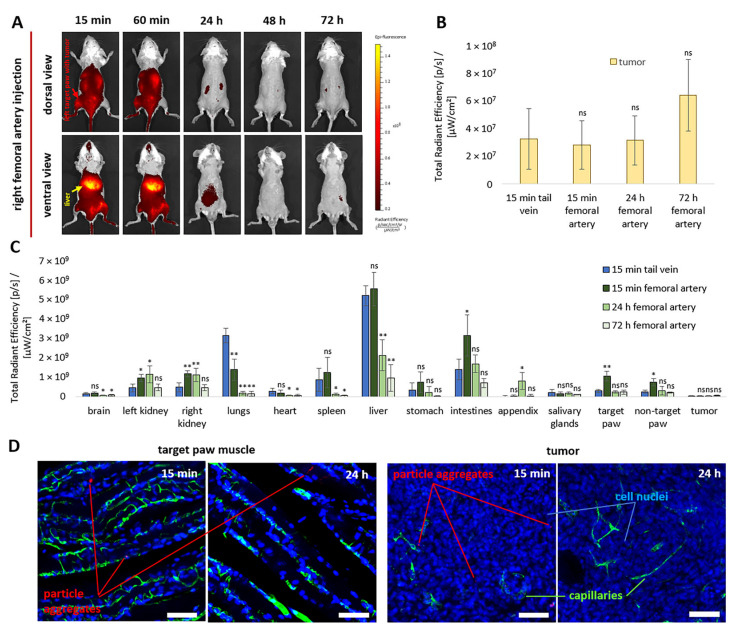
Fluorescence biodistribution dynamics during 72 h following right femoral artery injection of 2.5 × 10^10^ Cy7-labeled NPs in vivo (**A**) and ex vivo in the tumor (**B**) and other organs (**C**), compared with tail vein injection (*n* = 5 per each group). Representative confocal microscopy images of target muscle and tumor tissues 15 min and 24 h after NP injection. Blue indicates nuclei (DAPI), green indicates vessels (FITC–dextran, 500 kDa), and red indicates NP aggregates (Cy7) (**D**). Cryosection thickness: 70 µm; scale bar: 50 µm. All ex vivo data are presented as mean ± SD. Statistical analysis was performed relative to the control group (15 min tail vein injection) using an unpaired *t*-test with Welch’s correction. Statistical significance: ns, *p* > 0.05; *, *p* < 0.05; **, *p* < 0.01.

In vivo analysis of NP biodistribution dynamics following injection into the right femoral artery revealed accumulation of a bright fluorescent signal in the liver, with a decreasing trend over time. At the same time, fluorescence was also clearly detected in the left hind paw containing the tumor and in the ventral tail artery for at least 1 h (Figure 8A). Notably, this biodistribution pattern was not observed following intravenous administration (Figure A1) or injection via the carotid (Figure 4A) or renal (Figure 6A) arteries.

Ex vivo analysis of NP accumulation in tumor tissue revealed no significant differences between intravenous (15 min) and intra-arterial (15 min, 24 h, and 72 h) administration. Following intra-arterial delivery, fluorescence intensity in the target (left) and contralateral hind paws was 3.6-fold and 2.9-fold higher, respectively, than after intravenous injection at 15 min post-administration. However, this difference was no longer detectable at 24 h (Figure 8C).

Overall, femoral artery injection, similar to carotid artery administration, resulted in pronounced redistribution of fluorescence across organs compared with intravenous injection. This redistribution was reflected in increased fluorescence in the kidneys (2.4-fold), intestines (2.2-fold), and hind paws 15 min post-administration. Simultaneously, a reduction in off-target burden was observed in the lungs (2.3-fold), but not in the liver. During the subsequent 24 h, fluorescence intensity decreased in all organs and remained relatively high only in the liver, intestine, and appendix, indicating particle metabolism and elimination of the encapsulated dye via the gastrointestinal tract.

Confocal microscopic analysis of muscle and tumor cryosections revealed accumulation of isolated NP aggregates measuring 6–19 µm and 1–3 µm, respectively, predominantly within the capillary network 15 min after injection (Figure 8D). At the same time, aggregates were not detected at 24 h. The absence of irreversible occlusion of target muscle vessels is crucial for confirming the safety of the developed concept.

## 4. Discussion

Prolonged and controlled local drug release is an important task in biomedicine, as it can significantly reduce the side effects of systemic therapy [50,51,52]. This concept requires the selection of an optimal targeted drug delivery system, predominantly nanoscale, with the desired properties, which may also be useful for disease diagnosis and imaging [53,54,55]. Among biodegradable nanosized particles, polylactic acid (PLA) and polyglycolic acid (PGA) NPs and their copolymers (PLGA) occupy a special place [37,56,57]. They have been extensively studied, and numerous methods for their preparation have been developed [38,58,59]. In general, products made from these polymers are approved by regulatory authorities in different countries for implantation into the human body in the form of medical devices (sutures, grafts, implants) and therapeutic agents (polymer particles containing medicines) [60]. Nevertheless, PLA and PLGA particles providing prolonged drug release are currently approved as parenteral drug delivery systems for subcutaneous or intramuscular administration [61]. At the same time, their biocompatibility and high drug-loading capacity make them an attractive platform for creating nanoscale drug delivery vehicles for different routes of administration [62]. Modification of parameters such as the degree of polymerization, molecular weight of the copolymer, and the nature of polymer chain end groups enables the design of materials with tailored properties [63,64,65]. Moreover, in vitro release profiles can be fine-tuned by adjusting particle size and surface characteristics [57]. Nevertheless, the development of new drug carriers requires not only a comprehensive characterization of their physicochemical properties but also evaluation of their interactions with living systems and identification of efficient and safe in vivo delivery strategies to the desired site.

Currently, a key challenge is the accurate delivery of therapeutic NPs to the target organ or even to individual cells. The circulatory system represents a potentially universal route for delivering drug containers to almost any tissue or organ. Although the optimal particle size for different injection routes is still being actively discussed, particles in the 100–200 nm size range are generally considered effective for enhanced permeability and retention (EPR) in target tissues and tumors [66]. The synthesized PLGA NPs demonstrated good bio- and hemocompatibility in in vitro cell culture experiments (Figure 2F–I). No cytotoxic effects were observed for HEK 293, mMSCs, and C2C12 cell lines even after 48 h of incubation at a ratio of 8 × 10^6^ NPs per cell (Figure 2F). The viability of RAW 264.7 and L929 cell lines decreased by less than 12% under the same conditions. Good hemocompatibility was observed for WBCs and RBCs up to ratios of 1 × 10^4^ and 0.5 × 10^4^ NPs per cell, respectively (Figure 2H,I). The IC_50_ for leukocytes was not reached even at the highest tested dose, further supporting the potential of PLGA NPs as a drug delivery platform. It should be noted that the toxicity of PLGA NPs toward healthy cells reported by other authors largely depends on particle size and is primarily associated with the encapsulated cargo [47].

It is worth considering that, although the administered systemic dose is relatively low (based on the total circulating blood volume), the local concentration of NPs during the first pass through the artery can be extremely high and may potentially cause significant local hemolysis. Figure 2I shows hemolysis of nearly 32% at a ratio of 4 × 10^4^ NPs/RBC. Notably, this experiment reflects the incubation of a 1% RBC suspension with an equivalent volume of NPs (at the same concentration as that administered intra-arterially) for 1 h. Although the actual NP/RBC ratio in vivo is likely to be much lower and the exposure of RBCs to the full dose is much shorter, this potential risk should be taken into account and further investigated both in vitro and in vivo. This consideration is particularly important when incorporating a therapeutic agent into the NPs rather than a dye.

**Figure A5:**
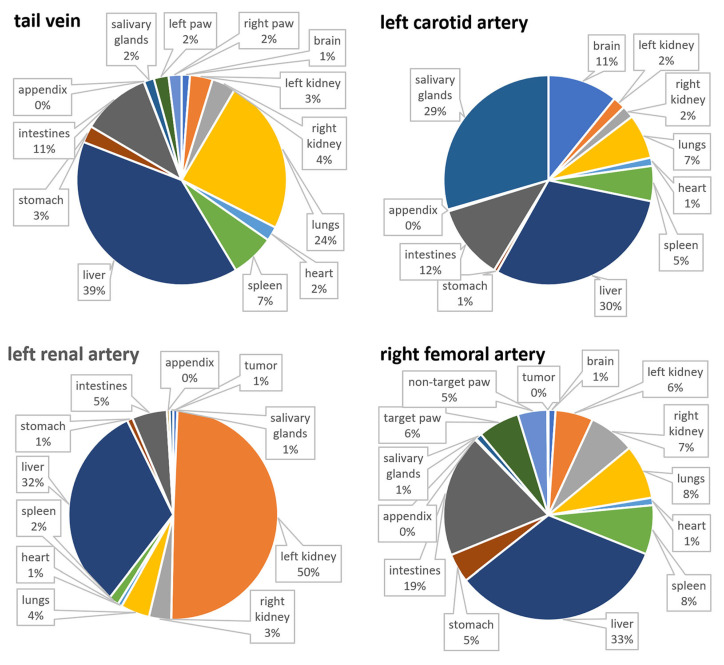
Ex vivo fluorescence biodistribution 15 min after injection of 2.5 × 10^10^ Cy7-labeled NPs *via* the tail vein (*n* = 5), left carotid artery (*n* = 6), left renal artery (*n* = 5), and right femoral artery (*n* = 5), expressed as a percentage of the total fluorescence detected across all examined organs and tissues.

As expected, intravenous administration of PLGA NPs resulted in predominant Cy7 accumulation in the lungs and liver (24% and 39% of total fluorescence, respectively) 15 min post-injection (Figure A1 and Figure A5). This distribution reflects the first-pass effect [6], as NPs are retained in pulmonary capillaries and subsequently cleared by hepatic Kupffer cells [67,68,69]. Consequently, intravenous delivery favors lung and liver accumulation of NPs, thereby complicating targeted delivery to other organs.

At the same time, the first-pass effect can be strategically exploited to deliver nano- and microcontainers to other organs and tissues when they are introduced into the bloodstream through the artery supplying the target region [15,18]. Nevertheless, this approach is not universally applicable to all drug carriers [33]. Therefore, delivery efficiency must be systematically evaluated for each new carrier type and arterial administration route.

Overall, arterial targeting of NPs via different arteries led to a pronounced shift in biodistribution compared with intravenous administration at 15 min (Figure 4, Figure 6, Figure 8 and Figure A5). Arterial delivery was effective across all investigated routes, increasing NP accumulation in the ipsilateral brain hemisphere (31.5-fold), salivary glands (28.3-fold), kidney (13.7-fold), and hind paw (3.6-fold), respectively, relative to intravenous injection.

Injection into the left carotid artery of 100 nm PLGA-NPs was accompanied by preferential accumulation of fluorescence in the brain (11%), salivary glands (29%), and liver (30%) (Figure A5). This accumulation pattern is most likely attributable to organ-specific vascular architecture, endothelial interactions governing NP adhesion and retention during first passage, and the presence of resident intravascular macrophages. Fluorescence accumulation in other examined organs and tissues ranged between 1% and 7%. The majority of the fluorescent signal detected in the brain was localized to the left (ipsilateral) hemisphere (Figure 4A,B). Accumulation in the ipsilateral hemisphere was 31.5-fold higher than that observed in the corresponding region following intravenous delivery (Figure 4B). The ability to achieve such efficient NP accumulation in brain tissue opens additional opportunities for localized modulation of blood–brain barrier (BBB) permeability, for example, through encapsulation and prolonged release of compounds that transiently increase BBB permeability [70,71]. Our data are consistent with findings reported by other authors for magnetic particles, liposomes, and cellular systems. For example, Tahara et al. investigated surface-modified PLGA NPs for carotid artery–mediated brain targeting using CLSM and demonstrated the high potential of this strategy [20]. However, delayed outcomes and procedural safety were not evaluated. Chertok et al. demonstrated that polyethyleneimine (PEI)-modified magnetic NPs (GPEI) can be effectively targeted to brain tumors via the carotid artery in combination with an external magnetic field [15], reporting an approximately 30-fold increase in tumor accumulation compared with intravenous administration. Cationic liposomes exhibited more efficient brain delivery after carotid artery injection than anionic or neutral liposomes [21]. Large cationic liposomes (~200 nm) demonstrated superior hemispheric and glioma targeting compared to smaller liposomes (60–80 nm) [22]. In contrast, Schackert et al. reported severe toxicity, presumably due to embolism, following intracarotid injection of 5-µm multilamellar liposomes [72]. They also observed no significant differences in accumulation between intravenous and intracarotid administration of smaller liposomes (1 μm or 40–80 nm). Finally, Zhang et al. demonstrated the potential of intra-arterial targeting of neural stem cells to the ipsilateral brain hemisphere via the carotid artery in mouse and rat ischemic stroke models [73], showing widespread distribution of injected GFP-labeled cells, preferential localization near ischemic lesions, long-term survival, and evidence of differentiation.

Injection of NPs via the renal artery demonstrated more effective delivery to the target organ than other intra-arterial routes. This effect may be attributed to the dense capillary network of the kidney and the presence of glomeruli with a highly complex capillary architecture (Figure A5). Preferential accumulation of NP-derived fluorescence was observed in the target kidney (50%) and liver (32%) (Figure A5). Accumulation of fluorescent signals in other examined organs and tissues did not exceed 1–5%. As expected, NPs accumulated predominantly in the renal glomeruli (Figure 6D), consistent with previous findings for polymer microcapsules 3–5 μm in size [25,26]. Notably, for this carrier type, comparable accumulation efficiency could not be achieved while preserving normal blood flow and tissue morphology. Other studies have also reported the potential of renal artery injection for the delivery of nanocarriers and cells to the kidney [24,74,75,76]. However, such studies remain scarce and rarely provide comprehensive safety or outcome assessments. Importantly, we also demonstrated a dose-dependent decrease in fluorescence intensity in the target kidney and observed no significant enhancement when increasing renal artery occlusion time after NP injection from 1 to 3 min. These findings are critically important for selecting appropriate dosing regimens for intra-arterial PLGA NPs delivery to the kidney.

Injection via the right femoral artery of PLGA NPs resulted in preferential accumulation of fluorescence in the liver (33%), intestines (19%), lungs (8%), and spleen (8%) (Figure A5). Moreover, 15 min post-injection, fluorescence intensity was 3.6-fold and 2.9-fold higher in the target (left) and contralateral (right) hind paws, respectively, compared with intravenous administration (Figure 8C). This corresponded to accumulation of approximately 6% of the total fluorescence in the target hind paw and 5% in the contralateral hind paw, relative to all examined organs and tissues (Figure A5). Nevertheless, this difference was no longer observed at 24 h (Figure 8A,C). The lower efficiency of NP accumulation in target hind paw muscle following femoral artery injection, compared with other intra-arterial routes, may be explained by the relatively low vascular density of muscle tissue compared with that of the brain and kidney. Notably, accumulation of PLGA NPs was still higher than that reported for magnetic microcapsules injected via the femoral artery [28], for which only a 1.5-fold increase in fluorescence intensity relative to intravenous injection was observed. In that case, application of an external magnetic field further enhanced accumulation by up to threefold.

Injection of NPs via the renal artery resulted in a 2.7–fold increase in the fluorescent signal in the subcapsular renal carcinoma graft compared with intravenous injection 15 min post-administration (Figure 6B). This corresponded to approximately 1% of the total fluorescence detected across all examined organs and tissues (Figure A5). At this time point (day 7), the tumor mass was only 20.6 ± 10.8 mg, corresponding to approximately 10% of the kidney mass and 0.001% of the total body mass of the mouse. In contrast, delivery of NPs via the right femoral artery did not result in a significant increase in fluorescence intensity in tumors induced by subcutaneous injection of CT26 cells into the left hind paw compared with intravenous administration (Figure 8B). This outcome may be associated with differences in tumor vascular architecture, including microvessel density, permeability, and vessel diameter [77,78]. At the same time, available literature does not allow direct comparison of vascularization dynamics between these tumor models, highlighting the need for further investigation in this area.

For all PLGA–NP administration routes (tail vein, carotid, renal, and femoral arteries), a bright fluorescent signal was consistently observed in target tissues and organs during the first hour, followed by a progressive decrease over 72 h (Figure A1A, Figure 4A, Figure 6A and Figure 8A). In addition, by 24 h, fluorescence was clearly visualized in the intestine and appendix, indicating gradual degradation of PLGA NPs with the release of encapsulated Cy7 under the action of resident tissue macrophages, particularly Kupffer cells. PLGA NPs also undergo hydrolytic degradation in the body, yielding CO_2_ and H_2_O, which are non-toxic and eliminated via the Krebs cycle [57]. Elimination of Cy7 through the gastrointestinal tract following systemic administration during carrier degradation is well documented and has been reported in other studies [79].

It is also important to note that arterial delivery of drug carriers may cause irreversible embolization of target organ vessels. Although delivery efficiency may be high under such conditions, this can lead to irreversible ischemic damage to the target tissue [30,72]. Such injury may result from inappropriately selected particle size [72], excessive dosage [26], or improper intra-arterial manipulation (Figure A2) [80]. Therefore, rigorous evaluation of both carrier parameters and minimally invasive surgical techniques is essential. In this study, we carefully monitored blood flow dynamics and tissue morphology to ensure that the proposed approach was both effective and safe (Figure 3, Figure 5 and Figure 7). Although confocal imaging revealed the presence of some NP aggregates in the brain (Figure 4D), muscle (Figure 8D), and renal glomerular capillaries (Figure 6D) 15 min after intra-arterial delivery, their size did not exceed 15 µm, and they were completely cleared from tissues within 24 h.

The spatial resolution of confocal microscopy did not allow reliable assessment of PLGA NP accumulation at the single-cell level in cryosections, due to the small particle size, difficulty distinguishing carrier fluorescence from tissue autofluorescence, and suboptimal dye excitation conditions. We hypothesize that PLGA NPs and their aggregates initially accumulate in target tissue vessels due to mechanical entrapment at vessel bifurcation and bends, rather than adhesion to endothelial cells. This is supported by the observation that aggregates were primarily located in these areas (Figure 4D) and within the complex capillary network of the kidney glomeruli (Figure 6D). Sobczynski et al. also demonstrated that PLGA particle entry into the bloodstream is accompanied by rapid formation of a protein corona, which reduces adhesion to vascular endothelial cells [81]. The absence of aggregates in the vasculature of target organs after 24 h also indirectly supports this hypothesis. We have previously observed a similar gradual decrease in the number of negatively charged polyelectrolyte microcapsules in the kidney glomeruli over time, most likely due to the gradual clearance of individual microcapsules and their aggregates [26]. Furthermore, the pronounced decrease in fluorescence across all organs by 24 h indicates an initial burst release of Cy7 (also noted in the in vitro experiment, Figure S2), high rate of nanoparticle degradation and metabolite clearance, most likely via hydrolysis [36] or Kupffer-cell-mediated metabolism in the liver [82]. Park et al. studied in detail the cellular distribution of PLGA NPs (size 271 ± 1.4 nm) in the mouse liver following in vivo administration [82]. They reported that Kupffer cells were the primary cells responsible for NP uptake, followed by liver sinusoidal endothelial cells and hepatic stellate cells. Only 7% of the NPs were detected in hepatocytes. Importantly, despite differences in initial NP biodistribution associated with the arterial targeting route, subsequent degradation and elimination kinetics followed a comparable pattern. Analysis of the percentage of the administered dose per gram of tissue (Figure S3) also revealed a trend toward a significant decrease in fluorescent signal in all organs over time, indicating gradual degradation of the carriers, release of the dye, and its elimination from the body.

Clinically, intra-arterial delivery is already a standard practice in interventional oncology (e.g., TACE). Taken together, the results presented here suggest that analogous 21strategies may be adapted for NP-based therapeutic platforms.

## 5. Conclusions

Minimally invasive endovascular surgery offers a wide range of solutions for controlled and localized drug delivery. This technique allows drugs to be administered with high precision through thin catheters, including direct delivery into small arteries supplying specific regions of the brain or other organs. It also enables temporary occlusion or redirection of blood flow. While arterial delivery still requires advanced equipment and highly skilled personnel, it opens up significant new possibilities for the local delivery of encapsulated drugs. The transient NP targeting effect likely does not, by itself, justify the use of minimally invasive surgery solely for arterial drug delivery. Nevertheless, this approach may become useful when drug delivery serves as an adjunct to a planned and unavoidable minimally invasive procedure, such as one performed for diagnostic purposes. It is also important to emphasize that this type of procedure does not require general anesthesia and is therefore accessible to a wider range of patients. In this context, targeting drug delivery systems via minimally invasive endovascular approaches represents a logical and promising strategy. At the same time, data regarding the post-administration fate of targeted drug delivery systems following intra-arterial delivery to different organs and tissues remain extremely limited. To date, this approach has been applied clinically primarily for irreversible chemoembolization of tumor-feeding arteries using cytotoxic agents and microspheres. The data presented in this study indicate that 100 nm PLGA NPs can serve as a versatile platform for effective and safe drug delivery to multiple organs and tissues. Administration of particles via the carotid, renal, and femoral arteries significantly increased NP-associated fluorescence in the brain, kidney, and hind paw, respectively. Comprehensive evaluation using blood flow monitoring, histological analysis, and confocal imaging of cryosections revealed no pathological changes associated with vascular occlusion or ischemic injury in the targeted organs and tissues. Taken together, these findings highlight the potential of the proposed strategy for prolonged local therapy of diverse pathological conditions affecting vital organs. Moreover, this approach demonstrates substantial translational potential, given the widespread clinical use of endovascular techniques across multiple fields of minimally invasive surgery. Nonetheless, anatomical and physiological differences between murine models and human patients must be carefully considered when extrapolating these findings. In particular, the impact of comorbid conditions such as atherosclerosis, varicose veins, or hypertension should be thoroughly evaluated when adapting these techniques for clinical application.

## Data Availability

The raw data supporting the conclusions of this article will be made available by the authors on request.

## Supplementary Materials

The following supporting information can be downloaded at: https://www.mdpi.com/article/10.3390/pharmaceutics18010085/s1, Information S1: Carotid artery catheterization; Information S2: Femoral and renal artery catheterization; Figure S1: NPs intensity and number-weight size distributions measured by dynamic light scattering; Figure S2: Dependence of the optical density at 760 nm on the concentration of Cy7 in plasma (A). Release of Cy7 from Cy7-PLGA NPs formulations (1, 24, 48, and 72 h) (B); Figure S3: Fluorescence biodistribution dynamics during 72 h following left carotid (A, *n* = 6), left renal (B, *n* = 5), and right femoral (C, *n* = 5) artery injection of 2.5 × 10^10^ Cy7-labeled NPs ex vivo. All ex vivo data are presented as % of administered dose/g (mean ± SD). Statistical analysis was performed relative to the experimental group—15 min artery injection—using an unpaired *t*-test with Welch’s correction. Statistical significance: ns, *p* > 0.05; *, *p* < 0.05; **, *p* < 0.01; Table S1: Summary of Cy7-PLGA NPs size distribution from DLS and SEM analysis.
