# Supplementary material for: Minimally Invasive Endovascular Administration for Targeted PLGA Nanoparticles Delivery to Brain, Salivary Glands, Kidney and Lower Limbs
Source: Pharmaceutics. 2026 Jan 9;18(1):85. doi: 10.3390/pharmaceutics18010085 (PMC12844752; doi:10.3390/pharmaceutics18010085)
Supplement: Supplementary file 1 [file pharmaceutics-18-00085-s001.zip › pharmaceutics-4002414-supplementary.pdf]

Supplementary Materials

Minimally Invasive Endovascular Administration for Targeted PLGA Nanoparticles Delivery to Brain, Salivary Glands, Kidney and Lower Limbs

Olga A. Sindeeva*, Lyubov I. Kazakova, Aleksandra Sain, Olga I. Gusliakova, Oleg A. Kulikov, Daria Terenteva, Irina A. Gololobova, Nikolay A. Pyataev, Gleb B. Sukhorukov*

Information S1

Carotid artery catheterization

Cy7 labeled NPs administration was performed by implanting a polyethylene cath-eter (PE – 10, Scientific Commodities INC., Lake Havasu City, Arizona) with thin polyu-rethane intravascular tubing on the end (40 mm, PU tubing, 32ga/.8Fr, 0.005 × 0.010 in, Instech) into the artery.

The mouse was positioned in dorsal recumbency. A small skin incision (2 – 3 mm) was made in the region above the salivary glands, which was then gently displaced to improve surgical access. Using fine tweezers, the carotid artery was carefully isolated from the adjacent structures, including the nerves and connective tissue. Throughout the procedure, the artery was intermittently moistened with saline to prevent desiccation and to minimize the risk of vessel fragility. Three temporary ligatures were applied to control blood flow to the carotid artery and minimize blood loss: 1 – closer to the heart (to block blood flow), 2 – to temporarily secure the catheter, and 3 – closer to the head (to block backflow of blood from the cerebral vessels). Blood flow into the artery was controlled by reducing the tension of upper or lower ligatures. A small puncture was created in the carotid artery wall using the tip of a syringe to facilitate catheter insertion. A thin segment of a saline – filled catheter (cut at a 45° angle) was then introduced into the vessel.

Once approximately 3 – 5 mm of the catheter was advanced into the artery, it was delicately secured using a medium ligature. Next, the upper ligature (located closer to the head) was loosened. Correct catheter placement was verified by gently retracting the sy-ringe piston connected to the catheter and confirming the arterial blood reflux. A 20 μL suspension containing 2.5 × 1010 NPs in saline was subsequently administered into the carotid artery at the specified dosage, considering the dead volume of the catheter (5 μL). The injection was performed slowly over 10 – 15 s to ensure uniform distribution of the suspension. Following the injection, the catheter was carefully withdrawn, and the arte-rial puncture site was sealed using a tissue adhesive. After complete polymerization of the adhesive, the ligatures were removed to restore blood flow. Finally, the surgical site was closed by suturing the skin.

Information S2

Femoral and renal artery catheterization

A detailed description of femoral and renal artery catheterization can be found in our protocol [28].

A polyethylene saline – filled catheter (PE – 10, Scientific Commodities INC., Lake Havasu City, Arizona) with thin polyurethane intravascular tubing on the end (40 mm, PU tubing, 32ga/.8Fr, 0.005 × 0.010 in, Instech) was introduced via the right femoral artery to a depth of 2 cm for femoral artery injection. For renal artery injection, items were pushed deeper and advanced into the abdominal aorta, up to the narrow and wide cath-eter (4.5 cm) section junction. During catheter insertion, proper placement was confirmed by gently retracting the syringe piston attached to the catheter and verifying the arterial blood backflow. The animal was then positioned in the left lateral recumbency, and a small longitudinal incision (8 – 10 mm) was made parallel to the spine, approximately 2 – 3 mm lateral to it, above the region of the left kidney. In the next stage, the junction of the left renal artery with the abdominal aorta was exposed by gently removing the sur-rounding adipose and connective tissue using cotton swabs to improve visibility. The aorta was probed with fine forceps to confirm the catheter position and locate its tip. The catheter was carefully retracted through the femoral access point until its tip was posi-tioned approximately 3 mm above the renal artery junction in the aortic lumen. Subse-quently, the catheter was gently inserted into the left renal artery. Limited blood flow into the kidney during the procedure was provided as the diameter of the renal artery slightly exceeded that of the catheter.

In the final phase of the surgery, a 20 μL suspension of 2.5 × 1010 Cy7 labeled NPs was injected into the arteries at a predetermined dose. The injection was performed over 10 – 15 s to promote the uniform distribution of the suspension throughout the vascula-ture. During this step, the renal artery walls were gently compressed around the catheter using forceps to prevent capsule reflux into the aorta while maintaining catheter patency. One minute post – injection, the forceps were released, the catheter was carefully with-drawn, and the femoral artery was ligated to prevent the hemorrhage. The muscle and skin layers were sutured to complete the procedure.

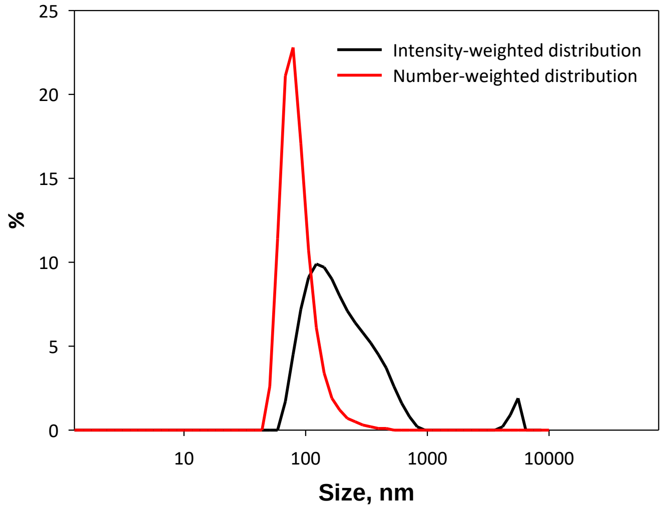

Figure S1. NPs intensity and number-weight size distributions measured by dynamic light scattering.

Table S1. Summary of Cy7-PLGA NPs size distribution from DLS and SEM analysis.

	DLS analysis	SEM
**PdI**	0.352	0.09
**Size distribution by Number ± SD, nm**	92.93 ± 37.65	–
**Size distribution by Intensity ± SD, nm**	215.7 ± 135.5	–
**Average size ± SD, nm**	–	89.54 ± 26.25
**Z-average, nm**	182.3	–

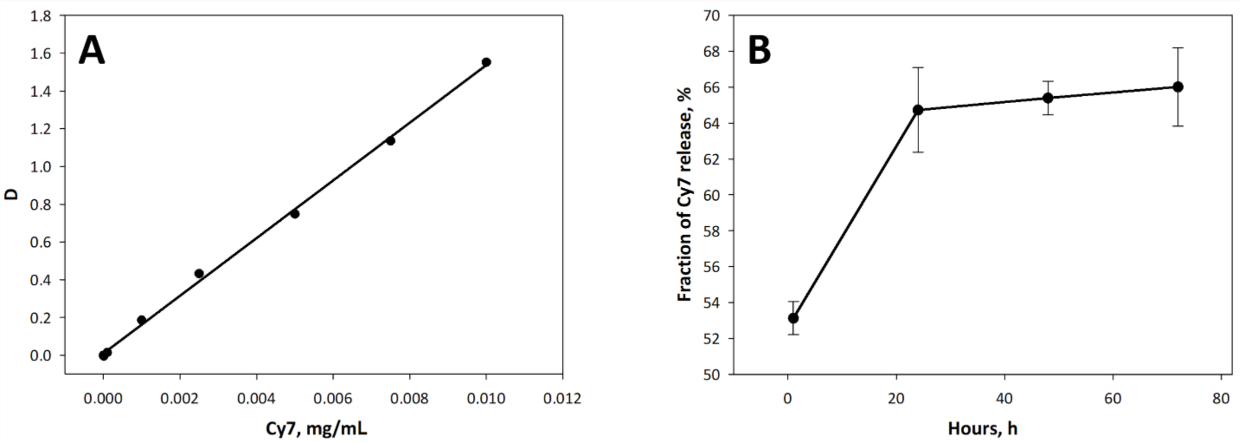

Figure S2. Dependence of the optical density at 760 nm on the concentration of Cy7 in plasma (A). Release of Cy7 from Cy7-PLGA NPs formulations (1, 24, 48, and 72 h) (B).

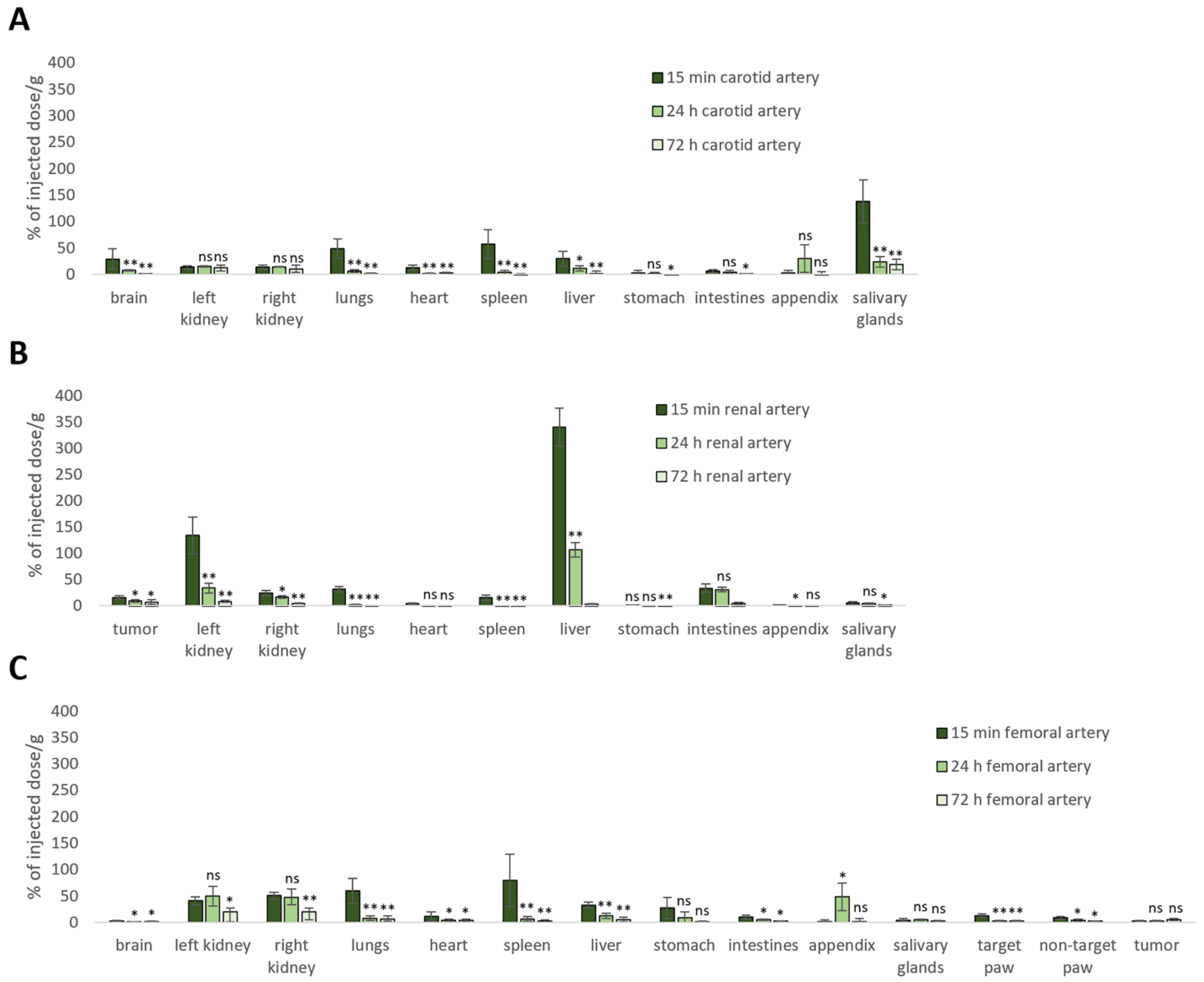

Figure S3. Fluorescence biodistribution dynamics during 72 h following left carotid (A, n=6), left renal (B, n=5), and right femoral (C, n=5) artery injection of 2.5×10^{10} Cy7-labeled NPs ex vivo. All ex vivo data are presented as % of administered dose/g (mean ± SD). Statistical analysis was performed relative to the control group (15 min artery injection) using an unpaired t-test with Welch's correction. Statistical significance: ns, $p > 0.05$; *, $p < 0.05$; **, $p < 0.01$.
